# Are hydrides under high-pressure–high-temperature superconductors?

**DOI:** 10.1093/nsr/nwad174

**Published:** 2023-06-19

**Authors:** J E Hirsch

**Affiliations:** Department of Physics, University of California, San Diego, USA

## Abstract

Contrary to the current consensus, I argue that the existing evidence for high-temperature superconductivity in hydrides under high pressure is **not** compelling. I suggest that the focus of the field should urgently shift to establish unequivocally experimentally whether or not superconductivity in pressurized hydrides exists, instead of continuing to search for new materials that might show elusive signals of unproven superconductivity at ever higher temperatures. The implications of a negative finding for the theoretical understanding of superconductivity are discussed.

## Teaser

The magnetic evidence for high temperature superconductivity in hydrides under pressure, when analyzed in its totality and considering its interrelationships, does not provide compelling evidence for superconductivity in these materials.

It has been eight years since the reported observation of conventional superconductivity above 200 K in sulfur hydride under pressure [1]. Since then, about 15 different hydride materials have been reported to be high-temperature superconductors. Conventional BCS-Eliashberg theory explains and in several cases (including [1]) predicted these observations, and hundreds more such compounds have been predicted theoretically. Room-temperature superconductivity would appear to be right around the corner.

But is this real? The field is entirely driven by theory, and, as a consequence, is subject to confirmation bias. When a sample predicted to be superconducting is found to show a drop in resistance, this is immediately interpreted as indicating superconductivity, ignoring the fact that there are other reasons why heterogeneous very small samples under enormous pressures could exhibit such drops [2]. Magnetic evidence that these materials are superconductors remains *scarce, spotty, contradictory and irreproducible*. Only for H_3_S and LaH_10_ does such evidence even exist. In what follows I discuss it and argue that it is far from compelling.

**Figure 1.**
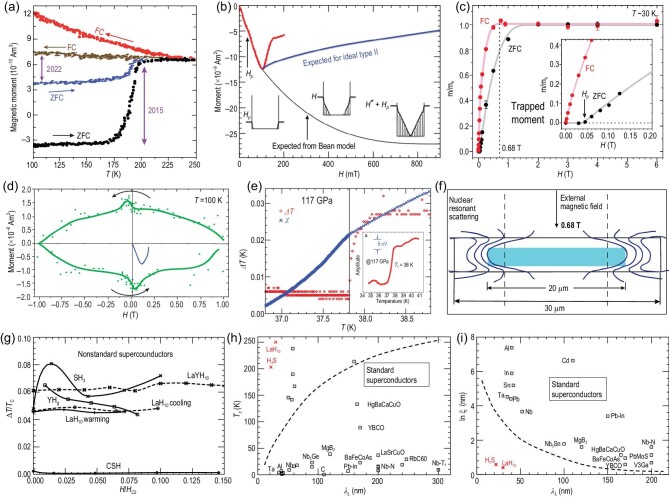
Examples that cast doubt on hydride superconductivity; see the discussion in the text. (a)–(f) are experimental results on sulfur hydride. (g) shows the width of the resistive transition versus magnetic field for various hydrides. (h) and (i) compare hydrides with other standard superconductors. (a): magnetic moment for applied magnetic field 20 Oe, adapted from Fig. 4 of Ref. [1] and Fig. S1 of Ref. [3]; (b): red curve adapted from Fig. 3 of Ref. [3]; (c): adapted from Fig. 2 of Ref. [6]; (d): green curve adapted from Fig. 4 of Ref. [14], blue curve adapted from Fig. 3 of Ref. [3]; (e): inset adapted from Fig. 2 of Ref. [16]; (f): adapted from Fig. S6 of Ref. [22]; (g): adapted from Fig. 11 of Ref. [29].

There is zero evidence for magnetic field *expulsion* under field cooling (FC), examples are shown in Fig. 1(a). While in some standard superconductors the effect can be very small for samples with strong pinning centers, there is no other class of known superconductors for which no evidence for field expulsion has been seen for any sample. Figure 1(a) also shows that the magnetic moment under zero-field cooling (ZFC) reported in 2022 [3–5] was approximately three times smaller than that reported in 2015 [1], for samples that were similar in diameter and thickness as estimated in [1,3]. Unlike under the FC protocol, under ZFC the measured signal is expected to depend only on sample volume and not on sample quality, casting doubt on the validity of these results.

The red curve in Fig. 1(b) shows reported diamagnetic moments versus magnetic field [3–5], indicating that magnetic fields smaller than 95 mT are excluded from the sample. For the same sample, magnetic fields as small as 45 mT are reported to penetrate and become trapped inside the sample when the applied field is removed [6], as shown in the inset of Fig. 1(c). This seems impossible; however, Minkov *et al.* [6] proposed to explain this anomaly by hypothesizing that the sample may have ragged edges that would allow penetration of the field even in the regime where the response of the sample is diamagnetic.

The presence of strong pinning centers is invoked to explain both the absence of signal under FC [3–5] and measurements of field trapping [6]. However, the rapid turnabout of the red curve in Fig. 1(b) beyond the field *H_p_* = 95 T interpreted as the lower critical field corrected for demagnetization [3–5] is inconsistent with the presence of strong pinning [7]. With strong pinning, the red curve should follow the behavior predicted by the Bean model [8], shown as the black curve in Fig. 1(b), or extensions of it [9,10], which describe the behavior seen experimentally in such materials that the magnetization magnitude continues to increase beyond the lower critical field where the magnetic field starts to penetrate the sample. At the very least, the magnetization magnitude should decay slower than what is expected for an ideal type-II superconductor with no pinning [11], shown as the blue curve in Fig. 1(b), which reaches zero only at the upper critical field. Also, the reported *linear* behavior of the trapped moment versus field in ZFC experiments [6] seen in the inset of Fig. 1(**c**) is in contradiction with the expected and observed *quadratic* behavior [12,13]. Also, the rapid decay of magnetization curves such as the red curve in Fig. 1(b) and the blue curve in Fig. 1(d) (also from [3]) is in direct contradiction with reported hysteresis cycles by the same authors [14], shown in Fig. 1(d): the blue curve should smoothly join the green curve. We note that the authors showed in Figure 6b of [15] a hysteresis loop extracted from measurements performed in 2015 after subtraction of a strong paramagnetic background where the virgin curve does smoothly join the hysteresis loop curve at magnetic field ∼300 mT. This is in stark contrast with the anomalous behavior seen in Fig. 1(d) measured seven years later [14] with presumably better samples and equipment.

Reported ac susceptibility measurements for H_3_S after background subtraction [16] are shown in the inset of Fig. 1(e), with the drop apparently indicating a superconducting transition. However, the underlying raw data, shown as the blue points in Fig. 1(e), merely show a kink at the presumed *T_c_*, which occurs at *precisely* the same temperature value where a change in the temperature interval at which the measurements were performed took place (red points in Fig. 1(e)) [17]. The same is seen at a different pressure [17]. This indicates to us that the superconducting ‘signal’ is an experimental artifact. Instead, in their reply [18] to our comment [17] the authors argued that ‘there are no relationships between the superconducting transition signals and those temperature breaks’, implying that in their view the coincidence seen in Fig. 1(e) between the kink in the blue symbols and the jump in the red symbols is merely a coincidence.

Struzhkin *et al.* [19] reported ac susceptibility measurements on LaH_10_; however, the signals are so weak and broad that it is impossible to draw any conclusions. The only other ac susceptibility measurements reported for a hydride material, CSH, were questioned in [20] and subsequently retracted [21].

The only other experiment reporting magnetic properties of a hydride indicating superconductivity is nuclear resonant scattering (NRS) [22,23]. That experiment reported that an applied magnetic field of 0.68 T was excluded from the interior of the H_3_S sample (Fig. 1(f)). That is in contradiction [24] with the magnetization measurements [3–5], as well as with flux trapping experiments [6]: for the geometry of [22], with demagnetizing factor 1/(1 − *N*) = 3.5, to explain the absence of the signal reported under applied magnetic field 0.68 T [22] would require that a three times larger magnetic field, i.e. 2.5 T, is excluded from the sample, in contradiction with the flux trapping experiments [6] that indicate that a magnetic field of ∼2 T fully penetrates and gets trapped (Fig. 1(c)). Furthermore, Fig. 1(c) shows that after applying a 0.68-T field and then removing it, $80\%$ of the maximum saturation moment remains trapped inside the sample, in contradiction with Fig. 1(f). Troyan *et al.* [25] argued that the NRS experiment [22] is consistent with the Minkov *et al.* [3–5] measurements if the critical current density is *J_c_* ∼ 6.8 × 10^7^ A/cm^2^; however, that is an order of magnitude larger than the critical current inferred by Minkov *et al.* from their measurements, *J_c_* ∼ 7 × 10^6^ A/cm^2^ [3–5].

Besides magnetic measurements, optical reflectance experiments were also claimed to show conventional superconductivity in sulfur hydride [26]. We requested the underlying raw data from the authors, and after analyzing them reported our conclusions to the authors and in a comment submitted and later published by the journal [27] that the published results did not reflect the raw data that were measured, nor did they provide evidence for conventional nor other superconductivity. Capitani *et al.* [26] published a reply to our comment [28], explaining that they had done corrections to the temperature-dependent background that were not explained in the paper because it is a standard procedure, and that they had obtained some of the published data from different raw data than those supplied to us. Readers should read our comment [27] and the authors’ reply [28] and draw their own conclusions.

From their magnetic measurements [3], Eremets and coworkers extracted values for the London penetration depth and coherence length for H_3_S and LaH_10_. The results are shown in panels (h) and (i) of Fig. 1 in red, compared with other known standard superconductors, both conventional and unconventional. It can be seen that the hydrides *strongly deviate* from the usual trends: for standard superconductors, small values of the London penetration depth are associated with low critical temperatures and type-I behavior (Fig. 1(h)), in stark contrast to the hydrides, and small values of the coherence length are associated with large values of the London penetration depth (Fig. 1(i)), in stark contrast to the hydrides. Finally, Fig. 1(g) shows [29] that the broadening of resistance curves observed in several hydrides does not increase with applied magnetic field, contrary to the usual behavior.

We should also point out that experimental results reported in this field are usually not reproduced by other researchers. For example, while Eremets’ group has reported zero resistance measurements for sulfur hydride [1], independent measurements by Nakao *et al.* [30] and by Osmond *et al.* [31] instead found that the resistance remains finite below the drop interpreted as the superconducting transition.

The conventional theory of superconductivity predicts unambiguously that hydrogen-rich materials under high pressure should be high-temperature superconductors [32], because their electron-phonon interaction is strong and their phonon frequencies are high, both effects contributing to high *T_c_*. What if these materials were ultimately found not to be superconductors, as suggested by the anomalies discussed in this perspective? What if the signals attributed to superconductivity were in reality due to other effects, namely, other physical phenomena or/and experimental artifacts? This would call into question the applicability of the conventional theory of superconductivity not only to hydrides but also to other materials [33].

I hope that experimentalists will urgently focus on determining unambiguously whether hydrides under pressure are or are not superconductors, unclouded by theoretical prejudices. This in my view is the greatest challenge facing experimentalists in the field today.
